# Author Correction: Microglial REV-ERBα regulates inflammation and lipid droplet formation to drive tauopathy in male mice

**DOI:** 10.1038/s41467-023-43360-6

**Published:** 2023-11-27

**Authors:** Jiyeon Lee, Julie M. Dimitry, Jong Hee Song, Minsoo Son, Patrick W. Sheehan, Melvin W. King, G. Travis Tabor, Young Ah Goo, Mitchell A. Lazar, Leonard Petrucelli, Erik S. Musiek

**Affiliations:** 1 Department of Neurology and Center On Biological Rhythms And Sleep, Washington University School of Medicine, St. Louis, MO USA; 2 Mass Spectrometry Technology Access Center at McDonnell Genome Institute (MTAC@MGI) at Washington University School of Medicine, St. Louis, MO USA; 3 Department of Neurology, Hope Center for Neurological Disorders, Knight Alzheimer’s Disease Research Center, Washington University School of Medicine, St. Louis, MO USA; 4 Institute for Diabetes, Obesity, and Metabolism, Perelman School of Medicine, University of Pennsylvania, Philadelphia, PA USA; 5 Department of Neuroscience, Mayo Clinic, Jacksonville, FL USA

**Keywords:** Alzheimer's disease, Microglia, Circadian mechanisms

Correction to: *Nature Communications* 10.1038/s41467-023-40927-1, published online 25 August 2023

The original version of this Article contained incorrect microscopy images in Figure 3f. In the original Figure 3, the top right panel immunofluorescence image in Figure 3f (labelled Cre+ and GFAP) was inadvertently duplicated from the top left panel of 3g (labelled Cre- and GFAP), and the lower right panel immunofluorescence image Figure 3f (labelled Cre+ and IBA1) was inadvertently duplicated from the lower left panel of 3g (labelled Cre- and IBA1).

The correct version of Figure 3 is:


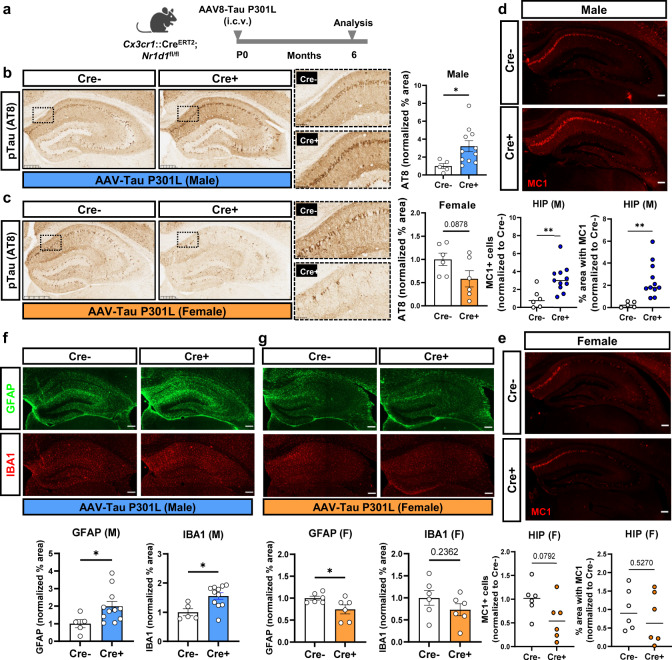


Which replaces the previous incorrect version:


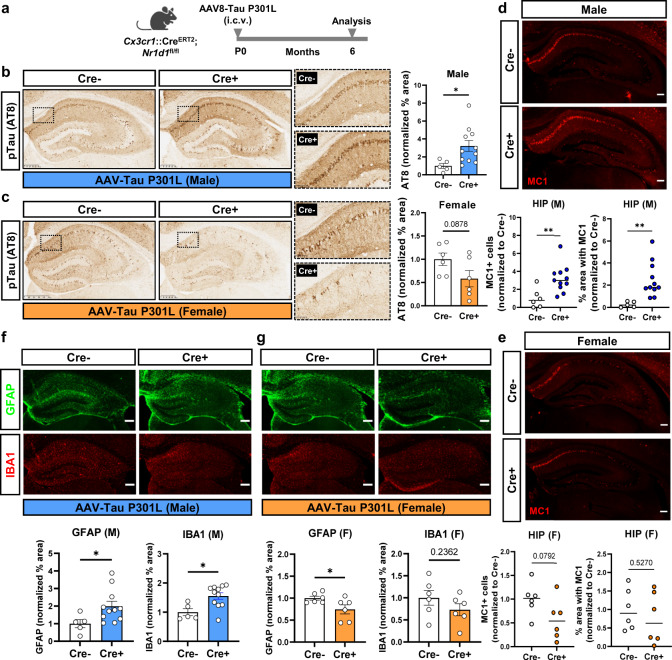


Figure 3 has been corrected in both the PDF and HTML versions of the Article.

