# Supplementary material for: Microglial REV-ERBα regulates inflammation and lipid droplet formation to drive tauopathy in male mice
Source: Nat Commun. 2023 Aug 25;14:5197. doi: 10.1038/s41467-023-40927-1 (PMC10457319; doi:10.1038/s41467-023-40927-1)
Supplement: Supplementary file 1 — Supplementary Information [file 41467_2023_40927_MOESM1_ESM.pdf]

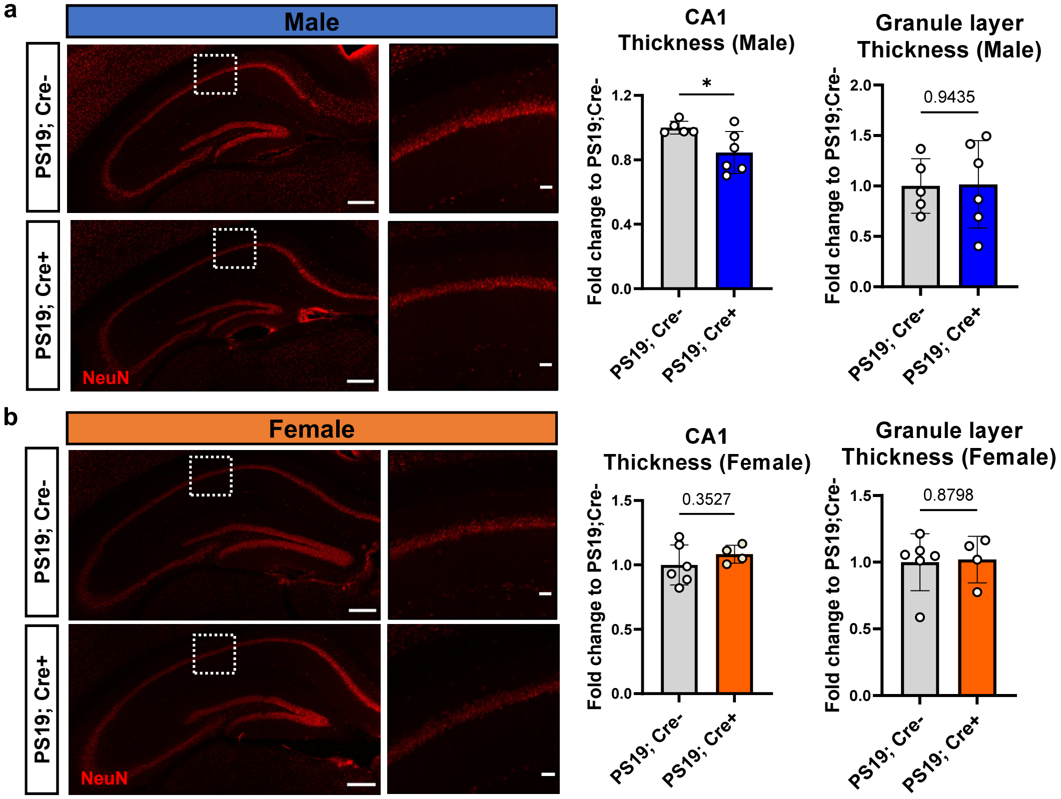

Supplementary Fig. S1: Neuronal loss in hippocampal CA1 region of male, but not female, microglial REV-ERBα KO PS19 mice. a, Hippocampal NeuN staining (dotted box, with inset) in male and **b,** female control P301S (PS19; Cre-) and microglial REV-ERBα KO P301S (PS19; Cre+). Each thickness of dentate gyrus granule and CA1 neuronal cell layers were presented by fold change (n=5-6 biologically independent mice). Scale bar, 500 μm (whole hippocampus), Scale bar, 50 μm (Zoom image). *p < 0.05 by 2-tailed T-test. P values >0.05 are listed. Error bars represent SEM.

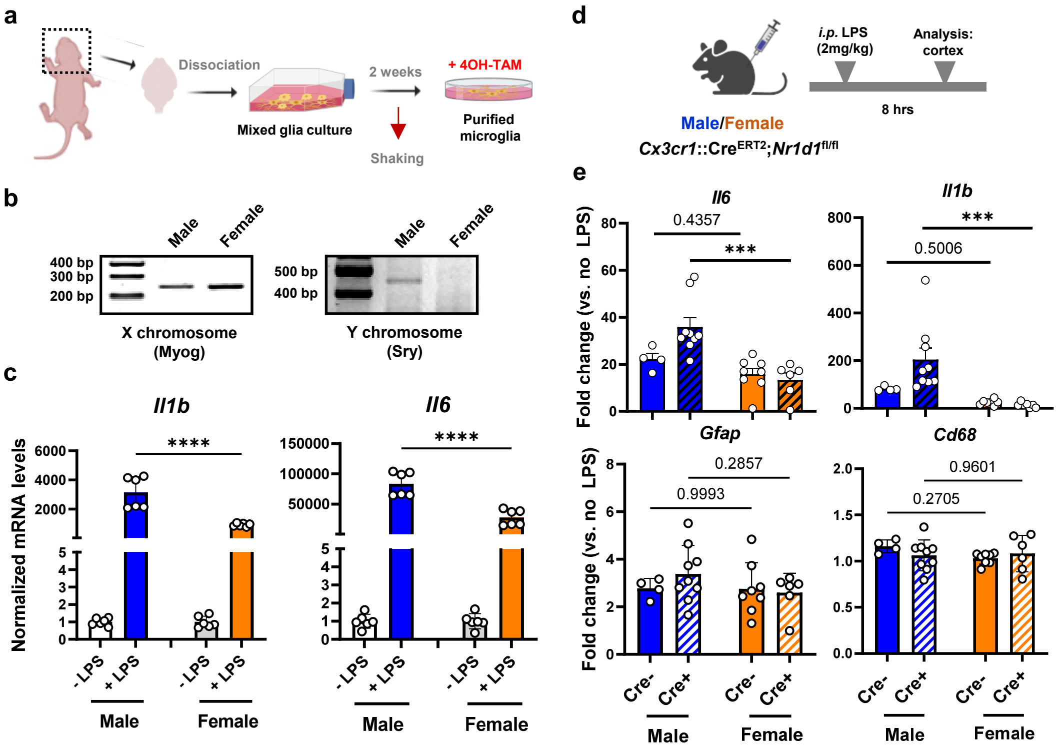

Supplementary Fig. S2: Male microglial REV-ERBα KO mice respond more robustly to LPS than female mice.

a, Procedure for preparing REV-ERBα KO cultured mouse microglia using CAG::Cre^{ERT2};*Nr1d1*$^{fl/fl}$ and Cre- littermate P1-3 pups. Microglia are treated *in vitro* with 1.5μM 4-hydroxyl-tamoxifen (4OH-TAM). Created with Biorender.com. **b,** Genetic sex determination of cultured microglia by PCR amplification of *Myog* (245bp) and *Sry* (441bp) for X-chromosome and Y-chromosome, respectively. **c,** Expression of pro-inflammatory cytokine transcripts including *Tnfα, Il6,* and *Il1b* in LPS-treated male and female cultured microglia (n=6, biologically independent samples). **d,** Experimental strategy for i.p. LPS in Cx3cr1::Cre^{ERT2};*Nr1d1*$^{fl/fl}$. Created with Biorender.com. **e,** Comparing the levels of pro-inflammatory cytokines (*Il6* and *Il1β*) and glial activation markers (*Gfap* and *Cd68*) between LPS injected WT (Cre-) and microglial REV-ERBα KO (Cre+) mice on both male and female in cerebral cortex (n=5-8 biologically individual mice). ***p < 0.005 and ****p < 0.001 by 2-way ANOVA with Sidak multiple comparisons test. P values >0.05 are listed. Error bars represent SEM.

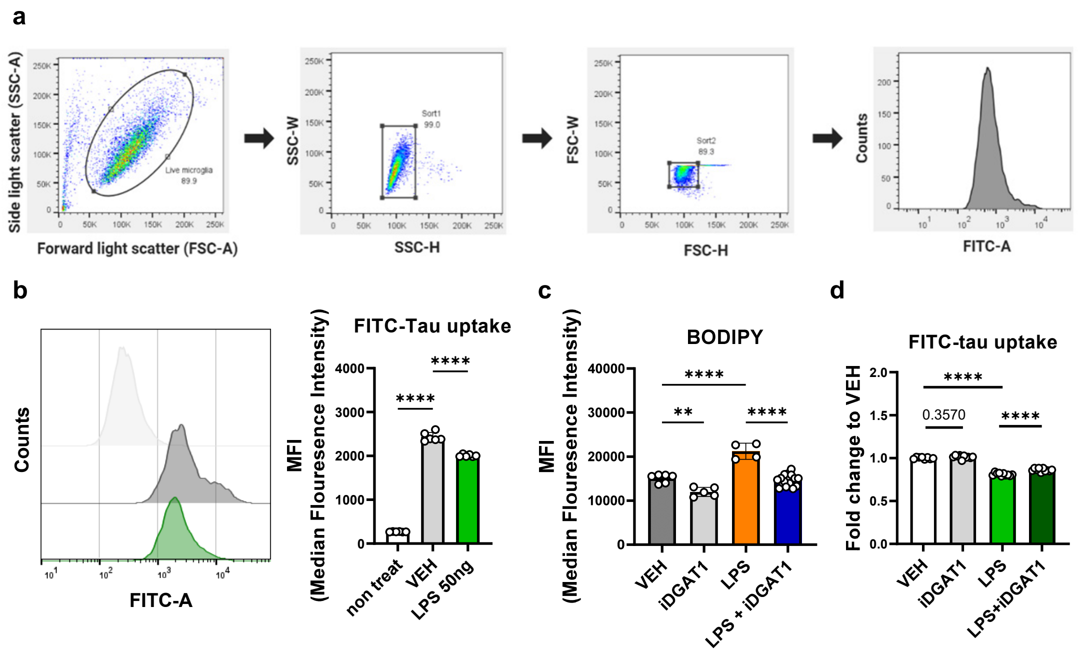

Supplementary Fig. S3: LPS-treated microglia shows defect of activity for internalization of tau. **a,** Gating strategies for Flow cytometry. Forward scatter (FSC-A) and Side light scatter (SSC-A) are proportional to particle size of live microglia and round analysis gate is further filtered twice with SSC-H/W and FSC-H/W parameters. Final population is analyzed by its fluorescence intensity. Data is collected from 10000 events. **b,** FITC-tau uptake assay in cultured microglia treated with VEH or 50ng/ml LPS for 24 hrs. FITC-tau signal was quantified by flow cytometry (n=6 from individual 6 pups). **c,** Increased BODIPY+ signal in LPS (50ng/ml) treated microglia was efficiently prevented by iDGAT1 (n=6-16 biologically individual samples) and **d,** dampened microglial tau uptake was also recovered (n=8-10 biologically individual samples). **p < 0.01 and ****p < 0.001 by one-way ANOVA or 2-way ANOVA with Sidak test or 2-tailed T-test. P values >0.05 are listed. Error bars represent SEM.

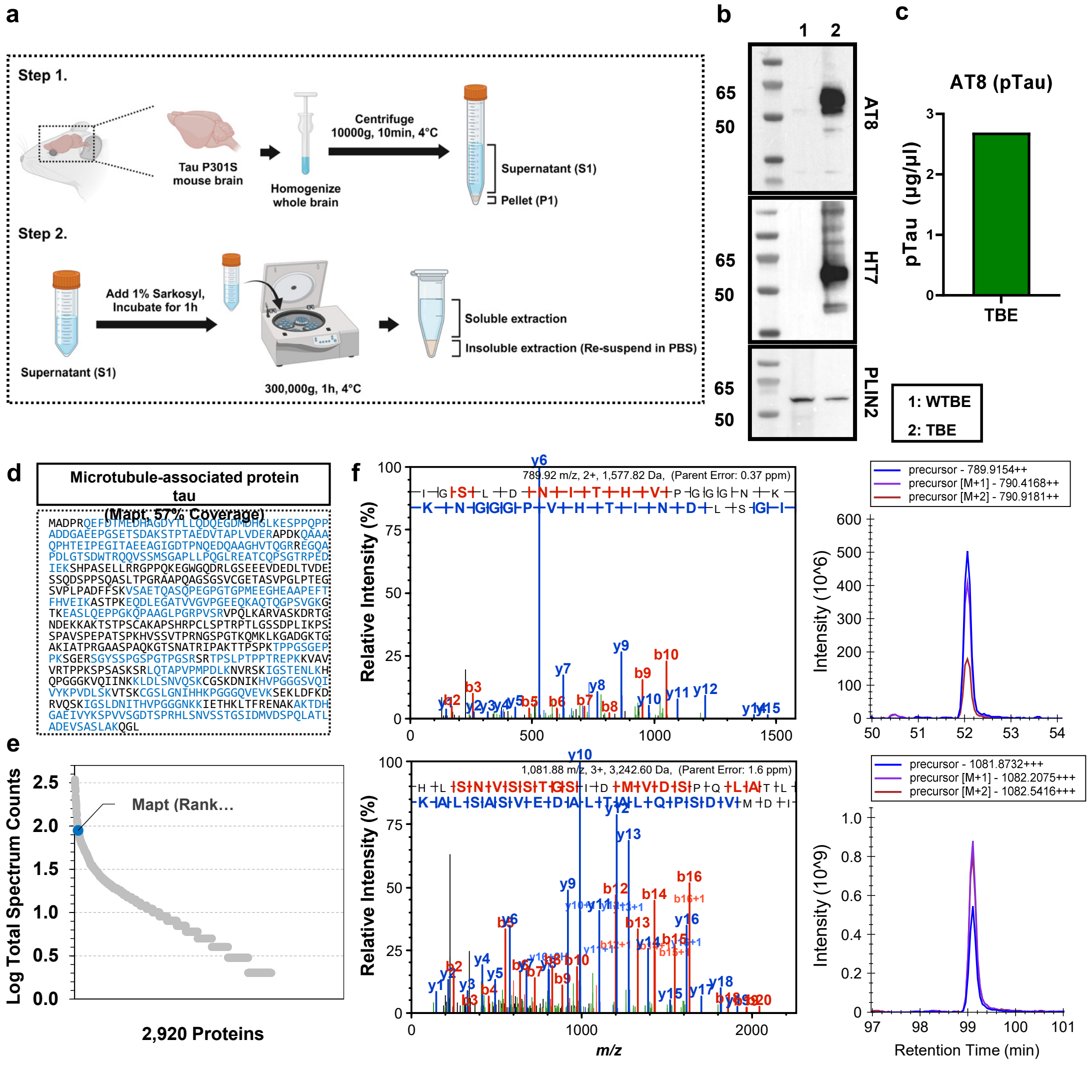

Supplementary Fig. S4: Generating Tau brain extract (TBE) which contains *in vivo* tau using aged PS19 mouse brain.

a, Schematic for generating Tau brain extract (TBE) using aged PS19 mouse brain. **b,** Abundant protein expression of total hTau (HT7), pTau (AT8), and PLIN2 in TBE. Wild-type brain extract (WTBE) does not contain as much tau (n=3 pooled from biologically independent samples). Created with Biorender.com. **c,** pTau levels in TBE by ELISA (2.7μg/μl). **d,** Protein sequence of Tau and coverage by mass spectrometry data. The peptides in blue were identified in the TBE sample. **e,** Dynamic range of TBE proteome. Tau protein (Mapt) hits 51st among the 2,920 identified proteins. **f,** MS/MS spectrum and peak intensity of Top 2 Tau peptides, 'IGSLDNITHVPGGGNK' and 'HLSNVSSTGSIDMVDSPQLATLADEVSASLAK'. y ions are in red and b ions are in blue, and the amino acids covered by the b- and y-ions are in color on the upper part of the MS/MS spectrum. The extracted ion chromatogram peak intensity of the three isotopic precursors are plotted.

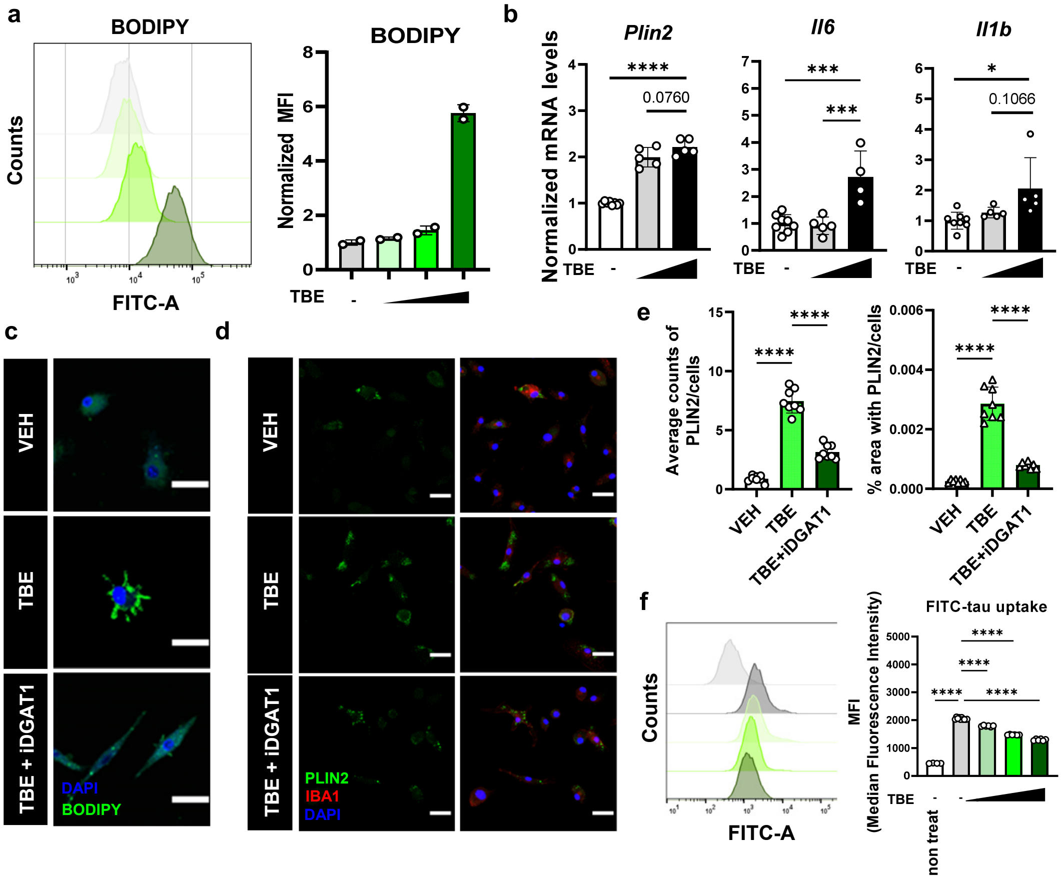

Supplementary Fig. S5: Characterization of the effects of TBE on microglial lipid droplet expression and tau uptake activity.

a, Dose-dependent changes in BODIPY+ cells (n=2 biologically individual samples) and **b,** the expression of *Plin2* and inflammatory transcripts (*Il6, Tnfa*, and *Il1b*) in TBE-treated cultured microglia (n=4-8 biologically individual samples). **c,** Images showing BODIPY+ lipid-droplet (LD) after TBE treatment in a cultured microglia and its prevention by LD blocker, iDGAT1 (A922500, 10μM). Green; PLIN2, blue; DAPI. **d,** Effect of TBE and LD blocker, iDGAT1 (A922500, 10μM), on PLIN2 protein expression in cultured microglia (green; PLIN2, red; IBA1, blue; DAPI) Scale bar, 50 μm. **e,** Quantified average counts and percentage of area of PLIN2 expression per cell (n=8 biologically individual samples). **f,** Dose-dependent attenuation of microglia-mediated internalization of FITC-tau aggregates by TBE treatment. Cells were incubated with TBE for 24 hours, washed, and then exposed to FITC-tau for 2 hours (n=4-10 biologically individual samples). *p < 0.05, **p < 0.01, and ****p < 0.001 by one-way ANOVA or 2-way ANOVA. P values >0.05 are listed. Error bars represent SEM.

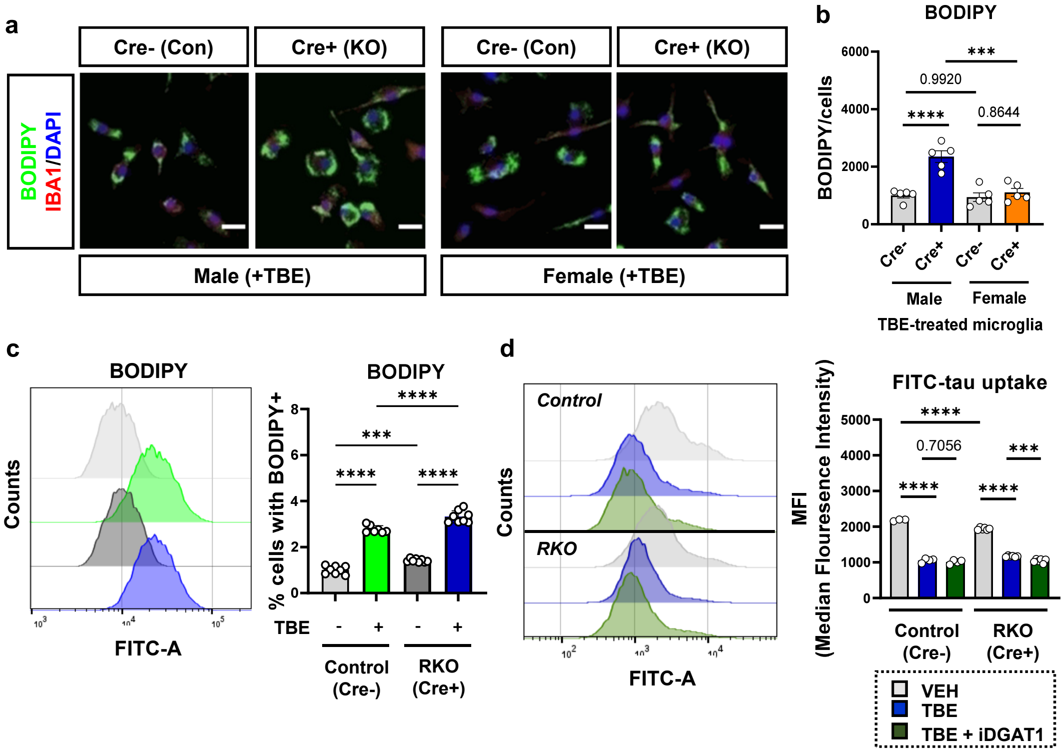

Supplementary Fig. S6: TBE amplifies lipid droplet expression in REV-ERBα KO microglia in a sex-dependent manner.

a, Representative images of BODIPY+ signal in TBE-treated male and female cultured microglia from Cre- control or Cre+ REV-ERBα KO mice (green; BODIPY, red; IBA1, blue; DAPI). Scale bar, 50 µm. **b,** Quantification of percentage of BODIPY+ signal from (a) (n=5 biologically individual samples). **c,** Percentage of BODIPY+ cells in male Cre- control and Cre+ REV-ERBα KO microglia cultures with or without TBE treatment, by flow cytometry (n=6-8 biologically individual samples). **d,** The effect of iDGAT1 on tau uptake in Cre- control or Cre+ REV-ERBα KO male microglia with or without TBE treatment (n=3-6 biologically individual samples). ***p < 0.005 and ****p < 0.001 by 2-way ANOVA with Sidak multiple comparisons test. P values >0.05 are listed. Error bars represent SEM.

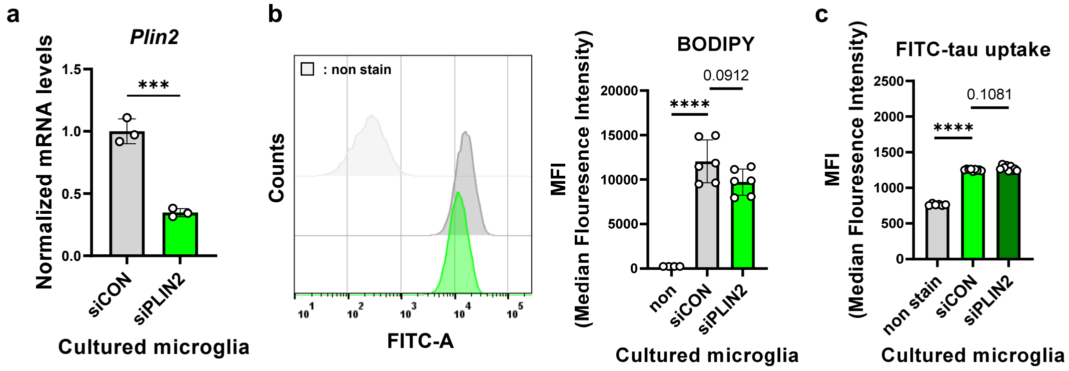

Supplementary Fig. S7: *Plin2* knockdown in cultured microglia does not affect tau uptake or LD accumulation.

a, Levels of *Plin2* mRNA in cultured microglia after treatment with control siRNA (siCON), or siRNA-targeting *Plin2*, normalized to control siRNA (n=3 biologically individual samples). **b,** Median Fluorescence Intensity (MFI) of BODIPY+ cells between siCon and siPlin2 cultured microglia (n=4-6 biologically individual samples). **c,** FITC-tau uptake in cells from a. (n=6-9 biologically individual samples). ***p < 0.005 and ****p < 0.001 by 1-way ANOVA with Sidak multiple comparisons test or 2-tailed T-test. P values >0.05 are listed. Error bars represent SEM.
