# Supplementary material for: Microglial REV-ERBα regulates inflammation and lipid droplet formation to drive tauopathy in male mice
Source: Nat Commun. 2023 Aug 25;14:5197. doi: 10.1038/s41467-023-40927-1 (PMC10457319; doi:10.1038/s41467-023-40927-1)
Supplement: Supplementary file 3 — Reporting Summary [file 41467_2023_40927_MOESM3_ESM.pdf]

nature portfolio

Corresponding author(s): Erik Musiek

Last updated by author(s): 8/2/2023

Reporting Summary

Nature Portfolio wishes to improve the reproducibility of the work that we publish. This form provides structure for consistency and transparency in reporting. For further information on Nature Portfolio policies, see our Editorial Policies and the Editorial Policy Checklist.

Statistics

For all statistical analyses, confirm that the following items are present in the figure legend, table legend, main text, or Methods section.

n/a	Confirmed	
☐	☒	The exact sample size (*n*) for each experimental group/condition, given as a discrete number and unit of measurement
☐	☒	A statement on whether measurements were taken from distinct samples or whether the same sample was measured repeatedly
☐	☒	The statistical test(s) used AND whether they are one- or two-sided<br>*Only common tests should be described solely by name; describe more complex techniques in the Methods section.*
☒	☐	A description of all covariates tested
☐	☒	A description of any assumptions or corrections, such as tests of normality and adjustment for multiple comparisons
☐	☒	A full description of the statistical parameters including central tendency (e.g. means) or other basic estimates (e.g. regression coefficient) AND variation (e.g. standard deviation) or associated estimates of uncertainty (e.g. confidence intervals)
☒	☐	For null hypothesis testing, the test statistic (e.g. F, t, r) with confidence intervals, effect sizes, degrees of freedom and P value noted<br>*Give P values as exact values whenever suitable.*
☒	☐	For Bayesian analysis, information on the choice of priors and Markov chain Monte Carlo settings
☒	☐	For hierarchical and complex designs, identification of the appropriate level for tests and full reporting of outcomes
☒	☐	Estimates of effect sizes (e.g. Cohen's d, Pearson's r), indicating how they were calculated

Our web collection on statistics for biologists contains articles on many of the points above.

Software and code

Policy information about availability of computer code

Data collection	QuantStudio 3 (Applied Biosystems) software was used to acquire qPCR data and images were collected by KEYENCE BZ-X800. BDX20 flow cytometry machine was used to acquire flow cytometry. For ELISA, kinetics were measured by Bio-Tek Synergy 2 plate reader and electron microscopy (EM) images were acquired on a TEM (Jeol JEM-1400 Plus). Orbitrap ID-X Tribrid Mass Spectrometer and Vanquish UHPLC (Thermo Scientific) were used for LC-MS and proteomics was performed by Vanquish Neo UHPLC System coupled to an Orbitrap Exploris 480 Mass Spectrometer Thermo Scientific). Sample RNA integrity was determined using a Tapestation and library preparation was performed by the SMARTer Ultra Low RNA kit for Illumina Sequencing (Takara-Clontech).
Data analysis	Prism 9 was used to perform statistical analysis on all data presented. All images and western-blot band analysis were performed by Image J 1.44 and flow cytometry data were analyzed and presented by FlowJo software (BD Biosciences). LC-MS related database search was performed by Compound Discoverer 3.3 with mzVault, mzCloud, and LipidBlast. For RNA-seq, basecalls and demultiplexing was performed with Illumina's RTA 1.9 software and the reads were aligned by STAR version 2.5.1a. Gene counts were quantitated with Subread:featureCount version 1.4.6-p5. All gene counts were then imported into the R/Bioconductor package EdgeR and adjusted TMM size factors and the matrix of counts were then imported into the R/Bioconductor package Limma.

For manuscripts utilizing custom algorithms or software that are central to the research but not yet described in published literature, software must be made available to editors and reviewers. We strongly encourage code deposition in a community repository (e.g. GitHub). See the Nature Portfolio guidelines for submitting code & software for further information.

Data

Policy information about availability of data

All manuscripts must include a data availability statement. This statement should provide the following information, where applicable:

- Accession codes, unique identifiers, or web links for publicly available datasets
- A description of any restrictions on data availability
- For clinical datasets or third party data, please ensure that the statement adheres to our policy

All data supporting this study are publicly available. Raw data from all figures can be found on Figshare: https://doi.org/10.6084/m9.figshare.23669112. The RNA-seq data generated in this study have been deposited in the Gene Expression Omnibus (GEO) under the accession code: GSE236875. [https://www.ncbi.nlm.nih.gov/geo/query/acc.cgi?acc=GSE236875]. We utilized Mus musculus Ensembl release 76 GRCm38 primary assembly for RNA-seq. Lipidomic analysis is also provided with this paper as a Source Data file and all other data are available upon request. Source data are provided as a Source Data file. The mass spectrometry proteomic data have been deposited to the ProteomeXchange Consortium via the PRIDE48 partner repository with the dataset identifier PXD043877 or at: https://www.ebi.ac.uk/pride/archive/projects/PXD043877. Human transcriptomic data is utilized by the data from AlzData web server [http://www.alzdata.org/download1.php], and the mouse ChIP-seq data sets are available in the NIH GEO repository under accession number GSE148644 or at https://www.ncbi.nlm.nih.gov/geo/query/acc.cgi?acc=GSE148644.

Human research participants

Policy information about studies involving human research participants and Sex and Gender in Research.

Reporting on sex and gender	N/A
Population characteristics	N/A
Recruitment	N/A
Ethics oversight	N/A

Note that full information on the approval of the study protocol must also be provided in the manuscript.

Field-specific reporting

Please select the one below that is the best fit for your research. If you are not sure, read the appropriate sections before making your selection.

☒ Life sciences ☐ Behavioural & social sciences ☐ Ecological, evolutionary & environmental sciences

For a reference copy of the document with all sections, see nature.com/documents/nr-reporting-summary-flat.pdf

Life sciences study design

All studies must disclose on these points even when the disclosure is negative.

Sample size	Sample size for mouse cohorts was determined based on sample size calculations using tau burden (AT8 staining % area) in the hippocampus as a primary endpoint, using previous data from the lab. Sample size calculation was designed to detect a 50% change in AT8 with 80% power and alpha=0.05. All samples sizes were described in each figure legends.
Data exclusions	No data were excluded.
Replication	Replicate experiments were successful and confirmed.
Randomization	The mice and cultured cells are naturally randomized based on sex and genotype.
Blinding	Third party animal staff with no knowledge of the mouse genotypes performed all mouse husbandry. During data preparation and analysis, mice are assigned a number and genotype is not indicated.

Reporting for specific materials, systems and methods

We require information from authors about some types of materials, experimental systems and methods used in many studies. Here, indicate whether each material, system or method listed is relevant to your study. If you are not sure if a list item applies to your research, read the appropriate section before selecting a response.

Materials & experimental systems

n/a	Involved in the study
☐	☒ Antibodies
☐	☒ Eukaryotic cell lines
☒	☐ Palaeontology and archaeology
☐	☒ Animals and other organisms
☒	☐ Clinical data
☒	☐ Dual use research of concern

Methods

n/a	Involved in the study
☒	☐ ChIP-seq
☐	☒ Flow cytometry
☒	☐ MRI-based neuroimaging

Antibodies

Antibodies used	Primary antibodies for IHC: mouse anti-NeuN (Sigma-Aldrich, MAB377, 1:200), mouse anti-GFAP (Novus, 5C10, 1:2000), rabbit anti-IBA1 (Wako, 019-19741, 1:1000), rat anti-CD68 (Bio-rad, MCA1957, 1:500), guinea pig anti-Plin2 (Fitzgerald, 20R-AP002, 1:200), biotinylated AT8 antibody (Thermo Fisher Scientific, MN1020, 1:1000)<br>Primary antibodies for WB: HT7 (Thermo Fisher, MN1000, 1:500), AT8 (Thermo Fisher, MN1020, 1:1000), and Plin2 (Abcam, ab52356, 1:1000).
Validation	All antibodies are commercially available and were commercially validated. We have validated all antibodies in our lab. GFAP, Iba1, and Cd68 have been validated by co-localization with other known glial antibodies. AT8, HT7, and MC1 have been previously validated by many labs, and we validated these by staining of tissue with no human tau transgene.

Eukaryotic cell lines

Policy information about cell lines and Sex and Gender in Research

Cell line source(s)	Primary mouse microglia-enriched cultures from sex-genotyped male and female P1-3 mouse pups.
Authentication	Staining of cultures for Iba1 and GFAP revelas that >90% of the cells are microglia (Iba1+, GFAP-)
Mycoplasma contamination	Cultures are tested regularly for mycoplasma; none detected.
Commonly misidentified lines<br>(See ICLAC register)	None used.

Palaeontology and Archaeology

Specimen provenance	*Provide provenance information for specimens and describe permits that were obtained for the work (including the name of the issuing authority, the date of issue, and any identifying information). Permits should encompass collection and, where applicable, export.*
Specimen deposition	*Indicate where the specimens have been deposited to permit free access by other researchers.*
Dating methods	*If new dates are provided, describe how they were obtained (e.g. collection, storage, sample pretreatment and measurement), where they were obtained (i.e. lab name), the calibration program and the protocol for quality assurance OR state that no new dates are provided.*

☐ Tick this box to confirm that the raw and calibrated dates are available in the paper or in Supplementary Information.

Ethics oversight	*Identify the organization(s) that approved or provided guidance on the study protocol, OR state that no ethical approval or guidance was required and explain why not.*

Note that full information on the approval of the study protocol must also be provided in the manuscript.

Animals and other research organisms

Policy information about studies involving animals; ARRIVE guidelines recommended for reporting animal research, and Sex and Gender in Research

Laboratory animals	Mice were used and were on a C57/Bl6J background. All mouse lines were obtained from Jackson Labs except Nr1d1(f/f) mice and are described in Methods. Nr1d1(f/f) mice were originally obtained from Dr. Mitchell Lazar at University of Pennsylvania, who generated this line. Mice were sacrificed at specific time based on the purpose from P1-3 (for microglial cultures) to 10 months of age (Tau expressing mice lines). All mice were housed under 12:12 hr light-dark cycle and free access to water and food. Multiple transgenic strains were used and are described in Methods.

March 2021

Wild animals	The study did not involve wild animals.
Reporting on sex	Sex was reported for all studies. We used both sex mice and cultured microglia which were sex-genotyped by markers of male (Sry) and female (Myog).
Field-collected samples	The study did not involve samples collected from the field.
Ethics oversight	All animal experiments were approved by the Washington University IACUC and were overseen by the WashU Dept. of Comparative Medicine, and all animal work complies with ARRIVE guidelines.

Note that full information on the approval of the study protocol must also be provided in the manuscript.

Dual use research of concern

Policy information about dual use research of concern

Hazards

Could the accidental, deliberate or reckless misuse of agents or technologies generated in the work, or the application of information presented in the manuscript, pose a threat to:

No	Yes	
☒	☐	Public health
☒	☐	National security
☒	☐	Crops and/or livestock
☒	☐	Ecosystems
☒	☐	Any other significant area

Experiments of concern

Does the work involve any of these experiments of concern:

No	Yes	
☒	☐	Demonstrate how to render a vaccine ineffective
☒	☐	Confer resistance to therapeutically useful antibiotics or antiviral agents
☒	☐	Enhance the virulence of a pathogen or render a nonpathogen virulent
☒	☐	Increase transmissibility of a pathogen
☒	☐	Alter the host range of a pathogen
☒	☐	Enable evasion of diagnostic/detection modalities
☒	☐	Enable the weaponization of a biological agent or toxin
☒	☐	Any other potentially harmful combination of experiments and agents

ChIP-seq

Data deposition

☐ Confirm that both raw and final processed data have been deposited in a public database such as GEO.

☐ Confirm that you have deposited or provided access to graph files (e.g. BED files) for the called peaks.

Data access links<br>*May remain private before publication.*	*For "Initial submission" or "Revised version" documents, provide reviewer access links. For your "Final submission" document, provide a link to the deposited data.*
Files in database submission	*Provide a list of all files available in the database submission.*
Genome browser session<br>(e.g. UCSC)	*Provide a link to an anonymized genome browser session for "Initial submission" and "Revised version" documents only, to enable peer review. Write "no longer applicable" for "Final submission" documents.*

Methodology

Replicates	*Describe the experimental replicates, specifying number, type and replicate agreement.*
Sequencing depth	*Describe the sequencing depth for each experiment, providing the total number of reads, uniquely mapped reads, length of reads and whether they were paired- or single-end.*

Antibodies	*Describe the antibodies used for the ChIP-seq experiments; as applicable, provide supplier name, catalog number, clone name, and lot number.*
Peak calling parameters	*Specify the command line program and parameters used for read mapping and peak calling, including the ChIP, control and index files used.*
Data quality	*Describe the methods used to ensure data quality in full detail, including how many peaks are at FDR 5% and above 5-fold enrichment.*
Software	*Describe the software used to collect and analyze the ChIP-seq data. For custom code that has been deposited into a community repository, provide accession details.*

Flow Cytometry

Plots

Confirm that:

☒ The axis labels state the marker and fluorochrome used (e.g. CD4-FITC).

☒ The axis scales are clearly visible. Include numbers along axes only for bottom left plot of group (a 'group' is an analysis of identical markers).

☒ All plots are contour plots with outliers or pseudocolor plots.

☒ A numerical value for number of cells or percentage (with statistics) is provided.

Methodology

Sample preparation	Cultured primary microglia were collected and further fixed by 4% PFA. BODIPY (BODIPY™ 493/503, Invitrogen) incubated with 1XPBS buffer on ice for 10 min and washed out for FACS analysis.
Instrument	Cells were analyzed using BDX20 FACS machine.
Software	FlowJo_v10.8.1 software (BD biosciences) for collection and GraphPad Prism 9 software for data analysis.
Cell population abundance	Cells that were stained with BODIPY were sorted by BDX20 FACS machine on EGFP expression. Cells were sorted using a standard two-way sort. Cells were determined to be >95% positive after sorting.
Gating strategy	Cells were gated on FSC/SSC (Mean FSC-A: ~10,000,000/Mean SSC-A: ~15,000,000). Cells were gated on FSC-H vs. Width to discriminate doublets. Positive cells were determined by gating on a negative (no BODIPY added) population.

☒ Tick this box to confirm that a figure exemplifying the gating strategy is provided in the Supplementary Information.

Magnetic resonance imaging

Experimental design

Design type	*Indicate task or resting state; event-related or block design.*
Design specifications	*Specify the number of blocks, trials or experimental units per session and/or subject, and specify the length of each trial or block (if trials are blocked) and interval between trials.*
Behavioral performance measures	*State number and/or type of variables recorded (e.g. correct button press, response time) and what statistics were used to establish that the subjects were performing the task as expected (e.g. mean, range, and/or standard deviation across subjects).*

Acquisition

Imaging type(s)	*Specify: functional, structural, diffusion, perfusion.*
Field strength	*Specify in Tesla*
Sequence & imaging parameters	*Specify the pulse sequence type (gradient echo, spin echo, etc.), imaging type (EPI, spiral, etc.), field of view, matrix size, slice thickness, orientation and TE/TR/flip angle.*
Area of acquisition	*State whether a whole brain scan was used OR define the area of acquisition, describing how the region was determined.*

Diffusion MRI ☐ Used ☐ Not used

Preprocessing

Preprocessing software	*Provide detail on software version and revision number and on specific parameters (model/functions, brain extraction,*

Preprocessing software | *segmentation, smoothing kernel size, etc.).*

Normalization | *If data were normalized/standardized, describe the approach(es): specify linear or non-linear and define image types used for transformation OR indicate that data were not normalized and explain rationale for lack of normalization.*

Normalization template | *Describe the template used for normalization/transformation, specifying subject space or group standardized space (e.g. original Talairach, MNI305, ICBM152) OR indicate that the data were not normalized.*

Noise and artifact removal | *Describe your procedure(s) for artifact and structured noise removal, specifying motion parameters, tissue signals and physiological signals (heart rate, respiration).*

Volume censoring | *Define your software and/or method and criteria for volume censoring, and state the extent of such censoring.*

Statistical modeling & inference

Model type and settings | *Specify type (mass univariate, multivariate, RSA, predictive, etc.) and describe essential details of the model at the first and second levels (e.g. fixed, random or mixed effects; drift or auto-correlation).*

Effect(s) tested | *Define precise effect in terms of the task or stimulus conditions instead of psychological concepts and indicate whether ANOVA or factorial designs were used.*

Specify type of analysis: ☐ Whole brain ☐ ROI-based ☐ Both

Statistic type for inference (See Eklund et al. 2016) | *Specify voxel-wise or cluster-wise and report all relevant parameters for cluster-wise methods.*

Correction | *Describe the type of correction and how it is obtained for multiple comparisons (e.g. FWE, FDR, permutation or Monte Carlo).*

Models & analysis

n/a	Involved in the study
☐	☐ Functional and/or effective connectivity
☐	☐ Graph analysis
☐	☐ Multivariate modeling or predictive analysis

Functional and/or effective connectivity | *Report the measures of dependence used and the model details (e.g. Pearson correlation, partial correlation, mutual information).*

Graph analysis | *Report the dependent variable and connectivity measure, specifying weighted graph or binarized graph, subject- or group-level, and the global and/or node summaries used (e.g. clustering coefficient, efficiency, etc.).*

Multivariate modeling and predictive analysis | *Specify independent variables, features extraction and dimension reduction, model, training and evaluation metrics.*
